# Whole-Genome Sequences of Two Manganese(II)-Oxidizing Bacteria, *Bosea* sp. Strain BIWAKO-01 and Alphaproteobacterium Strain U9-1i

**DOI:** 10.1128/genomeA.01309-16

**Published:** 2016-11-23

**Authors:** Kunihiro Okano, Seiko Furuta, Satoshi Ichise, Naoyuki Miyata

**Affiliations:** a Department of Biological Environment, Faculty of Bioresource Sciences, Akita Prefectural University, Shimoshinjo-Nakano, Akita City, Akita, Japan; b Lake Biwa Environmental Research Institute, Otsu, Shiga, Japan

## Abstract

This report describes the whole-genome sequences of two Mn(II)-oxidizing bacteria, filamentous Mn oxide microparticle-forming *Bosea* sp. strain BIWAKO-01 and alphaproteobacterium strain U9-1i.

## GENOME ANNOUNCEMENT

Microbial Mn(II) oxidation that yields insoluble Mn(III, IV) oxides is a fundamental biogeochemical process in terrestrial and aquatic environments (1–3). Bacterial Mn(II) oxidizers are widespread, yet their ecology remains an enigma (4, 5). For instance, filamentous Mn-rich microparticles that occur in stratified lake and marine environments are considered bacteriogenic, but this has not been clarified (5). Recently, an Mn(II)-oxidizing alphaproteobacterium, *Bosea* sp. strain BIWAKO-01 was found to produce filamentous Mn microparticles under laboratory conditions that are similar to those that occur naturally (6).

Strain U9-1i, isolated from a laboratory Mn(II)-oxidizing enrichment culture (7), is also an alphaproteobacterium, but it deposits formless Mn oxide phases, suggesting that physiological features of the two alphaproteobacteria are different. We temporarily named this alphaproteobacterium strain U9-1i, which is closely related to the *Caulobacteraceae* bacterium OTSz_A_272 (DDBJ/EMBL/GenBank accession no. CP013244) based on 16S rRNA gene sequence. Here, we report the whole-genome sequences of strains BIWAKO-01 und U9-1i.

Whole-genome sequencing of strain BIWAKO-01 was carried out using a PacBio RS II (Pacific Biosciences, Menlo Park, CA, USA) with a SMRTbell library (20 kbp). PacBio reads were assembled *de novo* using FALCON version 0.4.0, and draft contig errors were corrected by SMRTAnalysis version 2.3.0.140936.p4.150482.

Whole-genome sequencing of strain U9-1i was carried out using an Illumina HiSeq 1000 system (Illumina, San Diego, CA, USA), with a paired-end library (400 bp), and a Roche/454 PE Genome Sequencer FLX (454 Life Sciences, Branford, CT, USA), with a mate-pair library (8 kb). HiSeq reads were assembled *de novo* using Velvet version 1.2.08 and combined into a hybrid assembly with the 454 reads using GS De Novo Assembler version 2.8 (454 Life Sciences). Gaps between the 14 resulting contigs were closed using NESONI version 0.118 and *Platanus* version 1.2.1. The whole genomes were annotated using the RAST server.

The genomes of strains BIWAKO-01 and U9-1i were represented by two and five contigs (one scaffold), respectively. The strain BIWAKO-01 genome had a total length of 7,326,961 bp and a G+C content of 64.80%. It included 6,988 protein-coding sequences and 67 RNA-coding genes (i.e., four sets of rRNA genes and 55 tRNA genes). The annotation revealed that 4,938 coding sequences (CDSs) exhibited homology to genes with known functions, and the remaining 2,050 genes were identified as encoding hypothetical proteins of unknown function. The strain U9-1i genome had a total length of 4,053,247 bp and a G+C content of 63.30%. It included 4,196 protein-coding sequences and 48 RNA-coding genes (i.e., one set of rRNA genes and 45 tRNA genes). A total of 2,587 CDSs exhibited homology to genes with known functions.

Strain BIWAKO-01 had a multicopper oxidase (MCO) (locus_tag BIWAKO_02306 on accession no. BCQA01000001) with 78% sequence identity to MoxA, an Mn(II) oxidase of *Pedomicrobium* sp. (8). Our two strains also possessed proteins similar to MopA, a heme peroxidase-type Mn(II) oxidase (9–11): locus_tag BIWAKO_06830 of strain BIWAKO-01 and U91I_01921 of strain U9-1i, which were related to MopA (accession no. ABY99245) of *Pseudomonas putida* GB-1 (11) with 71% and 54% sequence identities, respectively. Strains BIWAKO-01 and U9-1i may use these enzymes for Mn(II) oxidation.

### Accession number(s).

This whole-genome shotgun project has been deposited at DDBJ/EMBL/GenBank under the accession numbers BCQA00000000 and BBSY00000000 and refers to the first versions that are described in this paper.
